# Effectiveness of Vaginal Pessary Use in Improving Quality of Life Among Women with Pelvic Organ Prolapse: A Prospective Study

**DOI:** 10.3390/healthcare13212659

**Published:** 2025-10-22

**Authors:** Ngoc Thi Tran, Thanh Quang Le, Hai Thanh Pham, Nam Hoang Tran

**Affiliations:** 1 General Planning Department, Tu Du Hospital, 284 Cong Quynh, Ho Chi Minh City 700000, Vietnam; 2 Tu Du Hospital, 284 Cong Quynh, Ho Chi Minh City 700000, Vietnam; 3 Research Center for Higher Education, Tokushima University, Tokushima 770-8502, Japan

**Keywords:** pelvic organ prolapse, pessary, quality of life, PFDI-20, PFIQ-7, conservative management, Vietnam

## Abstract

**Highlights:**

**What are the main findings?**

- Vaginal pessaries worked with high success, improvements in quality of life, and few side effects.
- Most women were satisfied and felt their symptoms improved.

**What are the implications of the main finding?**

- Pessaries can be used as an easy, safe first choice for women with prolapse.
- Offering pessary care in more hospitals could reduce the need for surgery.

**Abstract:**

Background: Pelvic organ prolapse (POP) significantly impairs women’s quality of life (QoL), particularly in resource-limited settings where surgical options may be restricted. Vaginal pessaries provide a conservative and cost-effective treatment, yet local evidence on their effectiveness in Vietnam remains scarce. Methods: In this six-month prospective study, 130 women with stage II–IV POP received vaginal pessaries. QoL was evaluated using validated PFDI-20 and PFIQ-7 questionnaires, and changes in symptoms, satisfaction, and adverse events were analyzed. Results: Most women presented with advanced POP (65.4% stage III, 19.2% stage IV). Ring pessaries were most frequently used (64.6%), followed by Gellhorn (23.9%) and Donut (11.5%). Successful fitting was achieved in 95.4% of participants, with six women discontinuing use due to expulsion or discomfort. QoL scores improved significantly after six months: mean PFDI-20 total decreased from 78.5 ± 51.4 to 42.2 ± 38.3 (*p* < 0.001), and PFIQ-7 total decreased from 62.6 ± 43.2 to 25.1 ± 22.9 (*p* < 0.001), with all subscales showing consistent improvement. Nearly all women (98.5%) reported symptomatic improvement, and 95.4% were satisfied with treatment. Correlation analyses showed no significant relationships between POP stage and obstetric factors (vaginal delivery, macrosomia, and episiotomy). In multivariate regression analysis including only age, BMI, and POP stage, none were significantly associated with QoL improvement. Conclusions: Vaginal pessary use was safe, highly effective, and well tolerated, leading to symptom and QoL improvements among Vietnamese women with advanced POP. These findings support pessary use as a first-line management option, especially for women who are elderly, have comorbidities, or lack access to surgery.

## 1. Introduction

Pelvic organ prolapse (POP) is a major gynecological condition arising from weakening of the pelvic floor support structures, leading to descent of the uterus, bladder, rectum, or vaginal vault into or beyond the vaginal canal. It represents one of the most common forms of pelvic floor dysfunction worldwide. According to the World Health Organization (WHO), pelvic floor disorders—including POP, urinary incontinence, and fecal incontinence—are increasingly recognized as significant contributors to morbidity and reduced quality of life among women, particularly in aging populations [1,2]. The global prevalence of POP has been estimated to range from 3% to 50% depending on diagnostic criteria, with symptomatic prolapse affecting approximately 10–20% of women [3]. The burden is projected to increase with rising life expectancy and higher survival after childbirth complications, especially in low- and middle-income countries where access to surgical care remains limited [2].

POP not only causes physical discomfort but also has profound psychosocial consequences. Women with POP often report urinary and bowel dysfunction, sexual difficulties, body image concerns, and restrictions in daily activities, all of which significantly impair overall well-being [4]. The condition is therefore not only a clinical problem but also a public health issue with wide-ranging social and economic implications. Addressing POP aligns with WHO’s strategic emphasis on improving women’s reproductive health and promoting healthy aging, highlighting the importance of effective, accessible, and culturally acceptable management strategies. Management strategies for POP range from conservative interventions to surgical repair, with treatment selection depending on severity, patient preference, comorbidities, and resource availability. Surgical procedures, including hysterectomy with suspension or reconstructive techniques, have long been regarded as the definitive option, particularly in high-income countries. However, growing concerns about surgical morbidity, recurrence, and mesh-related complications have led to re-evaluation of routine surgical management [5]. In parallel, the WHO and the International Federation of Gynecology and Obstetrics (FIGO) emphasize the need for accessible, cost-effective, and culturally acceptable conservative treatments to reduce global disparities in pelvic floor care [1,6].

Globally, there has been an increasing trend toward pessary use as a first-line therapy, particularly among older women, those desiring uterine preservation, or patients with significant surgical risk. In North America and Europe, over 70% of urogynecologists report using pessaries as initial treatment for POP [7,8,9]. In many low- and middle-income countries, especially in Sub-Saharan Africa, where surgical infrastructure and specialist services are limited, vaginal pessaries often serve as the only feasible option to manage pelvic organ prolapse. Studies from Uganda and Tanzania support this, showing improved outcomes and the feasibility of pessary therapy even when access to surgical care is constrained [10,11]. Moreover, the rising global focus on healthy aging and QoL has expanded the role of non-surgical approaches, positioning pessaries as an integral component of patient-centered POP management.

In Vietnam, evidence on pessary use remains limited to small case series and short-term observations, with no large prospective studies evaluating clinical and QoL outcomes using validated Vietnamese instruments. This gap hinders the development of national clinical guidance and patient counseling regarding nonsurgical management options. Pessaries were formally introduced into the management protocol for POP at Tu Du Hospital in 2012. Several local studies have evaluated their effectiveness. A study since 2012 reported a 94.7% success rate for pessary treatment, highlighting strict follow-up as a determinant of success [12]. Another study evaluated 184 women and found significant improvement in POP symptoms after 3 months of pessary use, though QoL was not assessed using standardized tools [13]. A prospective study of 77 women and demonstrated significant improvement in PFDI-20 and PFIQ-7 scores after 6 months, with a success rate of 90.9% [14]. More recently, a prospective study on 37 women reported an 81.1% improvement in QoL after 1 month, with adverse effects limited to minor vaginal discharge [15]. These findings indicate that pessary use is effective and well tolerated in the Vietnamese population, consistent with international evidence. However, most local studies were limited by small sample sizes, short follow-up, or lack of standardized QoL measures. The present study aims to contribute additional prospective data, with validated QoL instruments and a larger cohort, to further clarify the role of vaginal pessaries as a conservative treatment option for POP in Vietnam.

To our knowledge, this is the first large prospective study in Vietnam to evaluate vaginal pessary use for pelvic organ prolapse with validated Vietnamese versions of the PFDI-20 and PFIQ-7 instruments. These findings provide novel evidence of feasibility, effectiveness, and patient acceptability in a low-resource Southeast Asian setting.

## 2. Materials and Methods

### 2.1. Study Design and Participants

This study was designed as a prospective before–after interventional study without a control group. It was conducted at the Pelvic Floor Unit of Tu Du Hospital, from September 2022 to March 2023. Tu Du Hospital, the largest tertiary obstetrics and gynecology hospital in Vietnam, serves as a national referral center and provides a representative context for evaluating women’s health interventions [16]. The target population included women diagnosed with POP. Eligible participants were those with POP stage II or higher according to the Pelvic Organ Prolapse Quantification (POP-Q) system [17] who had an indication for pessary use and agreed to participate. Women were excluded if they had a history of hysterectomy or previous POP surgery, contraindications to pessary use such as genital tract infection, unexplained vaginal bleeding, or silicone allergy, or if they were unable to communicate in Vietnamese. Most participants were postmenopausal, and none reported current systemic or topical estrogen use. In Vietnam’s public healthcare setting, hormone replacement therapy is rarely prescribed for pelvic floor disorders, minimizing potential confounding effects of estrogen on vaginal tissue resilience or pessary tolerance. Menopausal status was therefore not included as a covariate in the multivariate analysis.

The required sample size was estimated using a paired comparison formula to detect significant differences in pre- and post-intervention quality-of-life scores. Key parameters included a two-sided test at a significance level of 0.05, as well as a statistical power of 80%, and based on expected effect sizes and variability derived from previously published data on pessary effectiveness in improving quality-of-life outcomes [18,19,20], a minimum of approximately 120 participants was required. To account for potential attrition, we recruited 152 women with symptomatic POP. Twenty-two were excluded due to exclusion criteria (10 with genital tract infection, 6 with prior POP surgery, 3 with silicone allergy, and 3 who declined participation). The remaining 130 participants were enrolled and fitted with pessaries. During the six-month follow-up, six participants (4.6%) discontinued pessary use because of discomfort or expulsion, but all completed the final assessment.

### 2.2. Procedures and Data Collection

At baseline, participants underwent structured interviews and clinical examinations. Demographic information, obstetric and medical history, and POP-Q staging were recorded. Details of pessary fitting, including type and size, were documented. Pessary fitting followed hospital protocol, and patients were instructed on care and scheduled for regular follow-up visits. Assessments were performed at baseline and at six months post-insertion to evaluate clinical and QoL outcomes.

Health-related QoL was measured using two validated instruments [19]: the Pelvic Floor Distress Inventory short form (PFDI-20) and the Pelvic Floor Impact Questionnaire short form (PFIQ-7). Both instruments had been translated, culturally adapted, and validated in Vietnamese populations, ensuring their reliability and relevance (Cronbach’s alpha coefficients above 0.80).

The PFDI-20 consists of 20 items divided into three subscales: the Pelvic Organ Prolapse Distress Inventory (POPDI-6), the Colorectal–Anal Distress Inventory (CRADI-8), and the Urinary Distress Inventory (UDI-6). Each question is scored from 0 (not at all) to 4 (quite a bit). Subscale scores are calculated as the mean response of answered items multiplied by 25, yielding a range of 0 to 100 for each subscale. The total PFDI-20 score is the sum of three subscales (range 0 to 300), with higher scores indicating greater symptom distress.

The PFIQ-7 contains 21 items grouped into three subscales: the Urinary Impact Questionnaire (UIQ-7), the Colorectal–Anal Impact Questionnaire (CRAIQ-7), and the Pelvic Organ Prolapse Impact Questionnaire (POPIQ-7). Each item is scored from 0 (not at all) to 3 (quite a bit). Subscale scores are calculated as the mean response multiplied by 33.3, resulting in a 0 to 100 range per subscale, and the total PFIQ-7 score is the sum of the three subscales (range 0 to 300). Higher scores reflect a greater impact of prolapse symptoms on daily activities. Decreases in PFDI-20 and PFIQ-7 scores represented improvements in symptoms and quality of life.

Patient-reported improvement was assessed by a direct question: “Do you feel your symptoms have improved after pessary use?”, with responses coded as “improved” or “not improved”. Satisfaction was defined as an affirmative response to the question, “Are you satisfied with the treatment?”. These subjective ratings were complemented by objective improvements in PFDI-20 and PFIQ-7 scores. Each questionnaire was administered at enrollment and at the six-month follow-up to assess changes in symptoms and functional impact. The reference period of three months minimized recall bias. To ensure consistency, three senior clinicians with certification in pelvic floor disorders conducted all interviews and resolved discrepancies by consensus.

Pessary management followed Tu Du Hospital’s standardized clinical protocol. All fittings were performed by trained urogynecologists. After sizing and comfort confirmation, the pessary was inserted and left in situ continuously. Patients were instructed not to remove the pessary at night. Follow-up examinations were scheduled at 1 month, 3 months, and 6 months after insertion. At each visit, the pessary was removed by the clinician for cleaning, vaginal inspection, and reinsertion after application of topical lubricant. Women were educated on perineal hygiene and instructed to report any discomfort, discharge, or expulsion immediately.

### 2.3. Statistical Analysis and Ethical Considerations

Data were entered into a secure database and analyzed using SPSS version 28.0 (IBM Corp., Armonk, NY, USA). Descriptive statistics were used to summarize participant characteristics. Normality of PFDI-20 and PFIQ-7 distributions was verified using the Shapiro–Wilk test. As both baseline and post-intervention scores met the assumption of normality, results are presented as mean ± standard deviation (SD). Associations between categorical variables, such as pessary type and POP-Q stage, were analyzed using the chi-square test. Paired statistical tests were applied to compare QoL scores before and after pessary use. Multivariate regression models were used to identify factors associated with improvements in QoL. A *p*-value of less than 0.05 was considered statistically significant.

The study was reviewed and approved by the Ethics Committee of Tu Du Hospital (2127/BVTD-HDDD on 10 November 2022). Written informed consent was obtained from all participants after detailed counseling on the objectives, procedures, potential benefits, and risks. Participation was voluntary, and women were free to withdraw at any time without affecting their standard medical care. Confidentiality was strictly maintained, and data were used solely for research purposes.

## 3. Results

### 3.1. Demographic Characteristics

A total of 130 women with symptomatic POP were included in the study. The mean age was 64.7 ± 7.3 years, and more than half of the participants were aged 60–69 years. The majority were of Kinh ethnicity and resided outside Ho Chi Minh City. Most participants had primary-school education or lower, and farming was the most common occupation (Table 1).

### 3.2. Obstetric History

Most participants (73.8%) experienced three or more deliveries, with the number of births ranging from 1 to 7 (median: 3). The majority (70.8%) reported at least three vaginal births. A history of episiotomy was common (71.5%), and 18.5% had delivered a macrosomic infant, with birth weights ranging from 2200 g to 4200 g. In the present study, we adopted a 3500 g threshold to define fetal macrosomia, in accordance with the local definition used in Vietnam [21]. This reflects the lower average birth weight of the population and evidence indicating that maternal and neonatal risks begin to increase above this level in Asian populations, rather than at the conventional 4000 g cutoff used elsewhere [22,23]. Cesarean section (CS) was less frequent, with only 8.5% having undergone the procedure (Table 2).

### 3.3. Clinical Characteristics and Pessary Fitting Outcomes

Most patients presented with advanced POP, predominantly stage III and IV according to the POP-Q classification. The ring pessary was the most frequently selected device, followed by Gellhorn and Donut types. The choice of pessary type was significantly related to the prolapse stage (χ^2^ = 11.4, *p* = 0.003), with Gellhorn pessaries more often used in women with stage IV prolapse, while ring pessaries predominated in stage II–III cases. Successful fitting was achieved in 95.4% of participants, with a small proportion being discontinued due to discomfort or expulsion (Table 3). Correlation analyses were performed to explore relationships between POP-Q stage and selected obstetric variables (number of vaginal deliveries, macrosomic birth, and episiotomy history). No significant correlations were observed between POP-Q stage and these variables (Spearman’s ρ ranging from 0.08 to 0.14, all *p* > 0.05). These variables were therefore excluded from subsequent multivariate regression models.

No major adverse events such as vaginal ulceration, infection, or bleeding were recorded during the six-month follow-up. Only minor complications, including discomfort and pessary expulsion, were observed and managed conservatively. Future studies should employ a structured adverse-event log to systematically quantify all complications and enhance the evaluation of long-term safety.

### 3.4. Quality of Life Outcomes

Substantial improvements in quality of life were observed after six months of pessary use. The mean PFDI-20 total score decreased from 78.5 ± 51.4 at baseline to 42.2 ± 38.3 (*p* < 0.001). All subscales of the PFDI-20 showed significant improvement: POPDI-6, CRADI-8, and UDI-6 (all *p* < 0.001). Similarly, the mean PFIQ-7 total score improved markedly, decreasing from 62.6 ± 43.2 to 25.1 ± 22.9 (*p* < 0.001). Each PFIQ-7 subscale (UIQ-7, CRAIQ-7, and POPIQ-7) also demonstrated significant reductions (all *p* < 0.001) (Table 4). When questionnaire outcomes were compared by POP-Q stage, no statistically significant differences were observed in baseline, post-intervention, or Δ-scores for either PFDI-20 or PFIQ-7 (all *p* > 0.05). When analyzed by subscale, the greatest reduction was observed in the Pelvic Organ Prolapse Distress Inventory (POPDI-6) domain, reflecting relief of pelvic pressure and bulging sensations. The Urinary Distress Inventory (UDI-6) also improved markedly, indicating fewer urinary incontinence and urgency complaints. The Colorectal–Anal Distress Inventory (CRADI-8) decreased, corresponding to improvement in bowel dysfunction such as constipation or fecal leakage. These findings demonstrate that pessary use provided multidimensional symptom relief across prolapse-related, urinary, and bowel domains.

### 3.5. Patient-Reported Improvement and Satisfaction

Patient-reported outcomes supported the objective findings. Almost all participants (98.5%) reported symptomatic improvement following pessary use, and 95.4% expressed overall satisfaction. Only two women (1.5%) reported no improvement, and six women (4.6%) were not satisfied with treatment (Table 5). When changes in QoL scores were compared by satisfaction status, women who reported satisfaction with pessary treatment had significantly greater improvements in both PFDI-20 and PFIQ-7 scores than those who were not satisfied (mean ΔPFDI-20: −38.2 ± 28.7 vs. −21.5 ± 19.4, *p* = 0.008; mean ΔPFIQ-7: −39.1 ± 27.2 vs. −24.8 ± 18.3, *p* = 0.012). This indicates that perceived satisfaction was associated with a larger reduction in symptom burden and functional limitation.

### 3.6. Factors Associated with Quality-of-Life Improvement

Multivariate regression analyses were conducted to identify predictors of change in QoL scores (ΔPFDI-20 and ΔPFIQ-7). Patient satisfaction was excluded from the model to avoid overlapping with dependent outcome measures. The final multivariate models include only age, body mass index (BMI), and POP-Q stage as independent predictors. For ΔPFDI-20, none of the examined variables—including age, BMI, or POP-Q stage—were significantly associated with improvement (Table 6). Similarly, for ΔPFIQ-7, no factor showed a significant independent association with outcome (Table 7).

## 4. Discussion

### 4.1. Main Findings

The present study adds novel contributions to the literature. It represents the first large prospective evaluation of vaginal pessary use in Vietnam using validated versions of the PFDI-20 and PFIQ-7. By enrolling 130 women—most with advanced prolapse—and demonstrating a 95.4% fitting success rate with significant improvements in symptom and quality-of-life scores, our findings provide robust local evidence to complement international studies. This methodological rigor and local focus address a key gap in the Southeast Asian context, where published data on pessary outcomes remain scarce.

This prospective study demonstrated that vaginal pessary use led to significant improvements in symptom burden and QoL among Vietnamese women with stage II–IV pelvic organ prolapse (POP). Nearly all participants experienced symptomatic relief, as reflected in marked reductions in PFDI-20 and PFIQ-7 scores across all subscales. The overall fitting success rate was high (95.4%), with only a small proportion discontinuing due to discomfort or expulsion. Importantly, almost all women reported subjective improvement (98.5%) and satisfaction (95.4%), confirming the acceptability of pessary treatment in this population.

### 4.2. Comparison with Previous Studies

Our findings align closely with international literature demonstrating that pessaries offer a safe, effective, non-surgical management strategy for pelvic organ prolapse (POP). For example, a French study reported satisfaction rates of 87.4% at six months after pessary fitting, alongside significant improvements in PFDI-20 and PFIQ-7 scores, very similar to the reductions we observed [24]. In Canada, more than 90% of women who continued pessary use reported improvements in QoL and symptom relief, with ease of use and comfort being major factors in satisfaction [25]. Beyond the overall reduction in total scores, domain-specific analyses highlight that prolapse-related, urinary, and bowel symptoms each improved substantially. This pattern mirrors international findings where pessary therapy reduced both urinary and fecal incontinence alongside prolapse discomfort, underscoring its broad functional benefits. Such multidimensional improvement strengthens the clinical relevance of pessary care as a conservative, non-surgical management option for women with advanced POP. These findings align with earlier literature showing that pessary therapy provides multidimensional symptom relief: not only for bulging or pelvic pressure but also for urinary incontinence/urgency and bowel dysfunction [26,27].

Meta-analyses and narrative reviews also support high fitting success rates. The International Urogynecology Consultation summarized that fitting success for vaginal pessaries across studies ranges from approximately 41% to 96.6%, with continued use rates likewise high [5]. These data are consistent with our findings of 95.4% fitting success and 95.4% patient satisfaction, reinforcing that even in more advanced POP, pessary therapy can perform very well. Further, in some studies, improvements in symptom burden measured by PFDI-20 and impact on daily life via PFIQ-7 were of the same order of magnitude as in our cohort. A study documented a significant reduction in PFDI-20 scores at six months (mean reduction of about 40–50 points depending on baseline severity), similar to our decrease from 78.5 to 42.2 [24]. These cross-study comparisons lend external validity to our results and suggest that vaginal pessary use yields robust symptom relief across different healthcare systems and patient populations.

In Vietnam, peer-reviewed evidence on pessary use is limited but points in the same direction as our findings: meaningful symptom relief and functional gains. A study from Can Tho University used the PFDI-20 and PFIQ-7 to evaluate women treated with a vaginal pessary, reporting reductions in symptom burden and improved QoL after short-term follow-up, consistent with our six-month improvements [15]. Broader Vietnamese [28,29] and worldwide [30,31] research on pelvic floor disorders has likewise adopted PFDI-20/PFIQ instruments and underscores quality-of-life deficits and barriers to care—patterns that pessary programs can address. While earlier local reports tended to be small cohorts with brief follow-up and heterogeneous outcome measures, our study adds to the national evidence base by pairing validated condition-specific instruments (PFDI-20, PFIQ-7) with a larger sample (N = 130) and six-month assessment—thereby providing more robust estimates of treatment effect and patient acceptability. The expanding use of PFDI-20/PFIQ-7 across Vietnamese urogynecology research also supports comparability with international literature and facilitates programmatic benchmarking [15,19,28].

These methodological advances enable us not only to confirm that symptom burden decreases substantially (as many studies show) but also that improvements in impact on daily life (as measured by PFIQ-7) are both clinically meaningful and long-lasting. This helps address prior criticisms in some local studies about transient or modest changes that may fade quickly.

### 4.3. Implications

Pessaries are especially valuable in resource-constrained contexts where access to surgical care is limited, or in populations with elevated surgical risk, such as elderly women and those with multiple comorbidities. Previous studies have highlighted that conservative management strategies, particularly pessaries, are cost-effective, safe, and feasible even in low- and middle-income countries [8,32]. The high success rate and minimal adverse events observed in our study reinforce pessary use as a first-line therapeutic option for Vietnamese women with POP, consistent with international findings demonstrating efficacy rates ranging from 41% to 96.6% [5]. Importantly, Vietnamese studies have similarly reported meaningful improvements in QoL after pessary use. For example, a study conducted at Tu Du Hospital showed significant reductions in PFDI-20 scores after short-term pessary placement [15], while another report at Can Tho University confirmed improvements using both PFDI-20 and PFIQ-7 instruments, despite smaller sample sizes and limited follow-up [28]. Our study builds on this local evidence with a larger cohort and six-month follow-up, adding robust data to support national practice. Furthermore, pessary provision can be effectively integrated into outpatient gynecologic services, as demonstrated in Canada and the UK, where pessary care reduced tertiary referrals and improved access for underserved populations [24,25]. Embedding pessary services within Vietnam’s outpatient system may therefore reduce the burden on tertiary hospitals while enhancing accessibility and continuity of care for women at the community level.

### 4.4. Predictors of Treatment Response

Multivariate regression analyses including age, BMI, and POP-Q stage as independent variables did not identify any significant predictors of quality-of-life improvement. Obstetric variables (vaginal delivery, macrosomia, and episiotomy) were analyzed separately and showed no significant correlation with POP-Q stage; they were therefore excluded from the regression model to avoid multicollinearity. This finding indicates that pessary use confers benefits across a wide spectrum of women, regardless of age, BMI, parity, or prolapse severity. Such universality has also been observed in international research. For example, symptom relief and satisfaction with pessary therapy were not strongly dependent on baseline stage or comorbidities [24], while similarly high levels of improvement across heterogeneous cohorts [25]. The absence of strong predictors in our study is consistent with Vietnamese reports [15,28].

In interpreting these findings, it is noteworthy that nearly all participants were postmenopausal, and none reported current use of systemic or topical estrogen. In Vietnam’s healthcare setting, menopausal hormone therapy remains uncommon, and hormonal treatment for pelvic floor disorders is rarely prescribed. This is consistent with regional evidence showing low acceptance of hormone therapy among Asian women [33] and with a 2023 Vietnamese study in which hormonal medication use was infrequent among midlife women [34]. Consequently, estrogen-related effects on vaginal tissue resilience or pessary tolerance were unlikely to confound our results. Future studies should document menopausal hormone therapy status and its potential influence on pessary outcomes.

Taken together, these findings reinforce the generalizability of pessary therapy as an effective first-line intervention. Nevertheless, the lack of identified predictors in our study may partly reflect limited statistical power and the omission of potentially relevant psychosocial variables. Factors such as partner support, sexual activity, cultural attitudes toward body image, and mental health have been shown to influence treatment satisfaction and adherence in pelvic floor disorders [8,32]. Incorporating these variables into future Vietnamese and regional studies will be crucial to provide a more holistic understanding of which patients are most likely to benefit from long-term pessary use.

### 4.5. Strengths and Limitations

This study has several notable strengths. It represents one of the largest prospective investigations of pessary use conducted in Vietnam to date, thereby contributing valuable local evidence to a literature base still dominated by Western data [15,28,30]. The use of validated Vietnamese versions of the PFDI-20 and PFIQ-7 ensures comparability with international studies and enhances the reliability of patient-reported outcomes [19]. Furthermore, the high follow-up rate at six months strengthens the internal validity of our findings, minimizing attrition bias that has challenged smaller studies in both Vietnam and other low-resource contexts.

Nonetheless, several limitations should be acknowledged. First, the single-center design may restrict generalizability to other healthcare settings, particularly in rural areas where gynecologic infrastructure and provider expertise are more limited. Second, the absence of a control group prevents direct comparisons with other management strategies, such as pelvic floor muscle training, surgical repair, or combined lifestyle interventions [32]. Third, while a six-month follow-up was sufficient to capture early improvements in symptoms and QoL, it may underestimate long-term discontinuation rates, late complications such as vaginal ulceration or infection, and possible changes in sexual function, which have been reported in longitudinal cohorts with follow-up extending beyond two years [24]. Finally, psychosocial variables such as marital support, cultural attitudes toward body image, and sexual activity were not assessed, limiting our ability to capture the full spectrum of factors influencing patient satisfaction and adherence. Future multicenter studies in Vietnam with longer follow-up durations, inclusion of control groups, and integration of psychosocial measures are warranted to provide a more comprehensive understanding of pessary outcomes.

### 4.6. Future Directions

Future research should prioritize multicenter studies with larger sample sizes to enhance external validity and capture variation across diverse healthcare settings in Vietnam, from tertiary hospitals to provincial and rural clinics. Longer-term follow-up is also essential to evaluate the durability of symptom relief, complication rates such as vaginal ulceration or infection, and continuation rates over multiple years—issues highlighted in international cohorts where discontinuation rates gradually increase beyond one year [5,24]. Comparative studies between pessary management and surgical options in the Vietnamese context are particularly warranted, as surgery remains the predominant treatment in urban centers, yet may be inaccessible or inappropriate for women with advanced age or comorbidities [8]. Randomized or pragmatic trials contrasting conservative and surgical strategies would provide valuable guidance for evidence-based policy and clinical decision-making.

In addition, qualitative research should be undertaken to explore patient perspectives on comfort, acceptability, and partner attitudes toward pessary use, as well as perceived barriers to uptake. International studies have shown that psychosocial and cultural factors—including stigma, partner support, and body image concerns—significantly shape adherence and satisfaction with pessary therapy [32,35]. Vietnamese studies have so far focused primarily on quantitative outcomes [15,28]; integrating qualitative methods would add critical insights to guide culturally sensitive interventions, patient counseling, and the development of training modules for gynecologic providers. Such mixed-methods approaches could ensure that pessary care in Vietnam is both clinically effective and socially acceptable, ultimately improving women’s access to conservative management options for pelvic organ prolapse.

## 5. Conclusions

This study confirms that vaginal pessary use is a safe, effective, and acceptable treatment option for Vietnamese women with advanced pelvic organ prolapse. Pessary therapy significantly improved symptoms and QoL, with high satisfaction rates and minimal complications. The benefits were observed across diverse demographic and clinical subgroups, underscoring broad applicability. These findings support the integration of pessary services into routine gynecologic practice in Vietnam as part of comprehensive, patient-centered management of pelvic organ prolapse. Expanding provider training, ensuring access to different pessary types, and strengthening patient education will be important steps to optimize outcomes and long-term adherence.

## Figures and Tables

**Table 1:** Demographic characteristics of study participants (N = 130).

Characteristic	*n*	%
Age (mean ± SD)	64.7 ± 7.3	
<60 years	29	22.3
60–69 years	69	53.1
≥70 years	32	24.6
Occupation		
Farmer	42	32.3
Worker	18	13.9
Trader	16	12.3
Government employee	7	5.4
Housewife	19	14.6
Other	28	21.5
Ethnicity		
Kinh	123	94.6
Other	7	5.4
Residence		
Ho Chi Minh City	22	16.9
Other provinces	108	83.1
Education		
Below primary	15	11.5
Primary school	72	55.4
Secondary school	24	18.5
High school	14	10.8
College/University	5	3.8

**Table 2:** Obstetric history of participants (N = 130).

Variable	*n*	%
Number of births < 3	34	26.2
Number of births ≥ 3	96	73.8
Vaginal deliveries < 3	38	29.2
Vaginal deliveries ≥ 3	92	70.8
Episiotomy—Yes	93	71.5
Episiotomy—No	37	28.5
Cesarean section—Yes	11	8.5
Cesarean section—No	119	91.5
Macrosomic birth ≥ 3500 g	24	18.5
No macrosomic birth	106	81.5

Note: “Number of births” refers to total live births per participant regardless of delivery mode. “Vaginal deliveries” indicate the number of births delivered vaginally (participants with both vaginal and cesarean deliveries are included in both categories). “Cesarean section” refers to whether a participant had ever delivered by cesarean. Macrosomic birth refers to a birth weight ≥ 3500 g.

**Table 3:** Clinical characteristics and pessary fitting outcomes (N = 130).

Characteristic	*n*	%
POP-Q stage II	20	15.4
POP-Q stage III	85	65.4
POP-Q stage IV	25	19.2
Ring pessary	84	64.6
Gellhorn pessary	31	23.9
Donut pessary	15	11.5
Successful fitting	124	95.4
Discontinued (discomfort/expulsion)	6	4.6

**Table 4:** Changes in PFDI-20 and PFIQ-7 scores before and after 6 months of pessary use (N = 130).

Instrument/Subscale	Baseline (Mean ± SD)	6 Months (Mean ± SD)	Δ (Post–Pre) (Mean ± SD)	*p*-Value
PFDI-20 total	78.5 ± 51.4	42.2 ± 38.3	−36.3 ± 29.1	<0.001
POPDI-6 (prolapse)	31.4 ± 21.1	15.1 ± 11.7	−16.3 ± 10.2	<0.001
CRADI-8 (bowel)	13.6 ± 8.5	8.2 ± 6.1	−5.4 ± 3.8	<0.001
UDI-6 (urinary)	33.5 ± 18.9	17.9 ± 15.2	−15.6 ± 12.0	<0.001
PFIQ-7 total	62.6 ± 43.2	25.1 ± 22.9	−37.5 ± 27.6	<0.001
UIQ-7	28.4 ± 19.4	11.6 ± 7.9	−16.8 ± 12.5	<0.001
CRAIQ-7	14.6 ± 12.4	7.7 ± 2.3	−6.9 ± 7.2	<0.001
POPIQ-7	19.6 ± 15.5	5.8 ± 1.6	−13.8 ± 9.3	<0.001

Values are expressed as mean ± SD. Δ represent the mean change in total score (post–pre intervention). Negative Δ values indicate improvement (score reduction). Normality was verified using the Shapiro–Wilk test; all data were normally distributed.

**Table 5:** Patient-reported improvement and satisfaction after 6 months of pessary use (N = 130).

Patient-Reported Outcome	*n*	%
Improved	128	98.5
Not improved	2	1.5
Satisfied	124	95.4
Not satisfied	6	4.6

**Table 6:** Multivariate regression analysis of factors associated with ΔPFDI-20 score (N = 130).

Predictor Variable	β Coefficient (95% CI)	*p*-Value
Age	−0.16 (−5.7 to 0.5)	0.106
Body mass index (BMI)	0.17 (−0.1 to 8.2)	0.062
POP-Q stage	0.05 (−3.8 to 5.8)	0.683

Multivariate linear regression was performed with ΔPFDI-20 as the dependent variable. CI = Confidence Interval.

**Table 7:** Multivariate regression analysis of factors associated with ΔPFIQ-7 score (N = 130).

Predictor Variable	β Coefficient (95% CI)	*p*-Value
Age	−0.08 (−4.5 to 1.7)	0.386
Body mass index (BMI)	0.11 (−1.2 to 5.1)	0.222
POP-Q stage	0.08 (−3.2 to 6.2)	0.538

Multivariate linear regression was performed with ΔPFDI-7 as the dependent variable. CI = Confidence Interval.

## Data Availability

Data is unavailable due to privacy and ethical restrictions.

## Abbreviations

The following abbreviations are used in this manuscript:

BMI	Body Mass Index
CI	Confidence Interval
CRADI-8	Colorectal–Anal Distress Inventory-8
CRAIQ-7	Colorectal–Anal Impact Questionnaire-7
CS	Cesarean Section
FIGO	International Federation of Gynecology and Obstetrics
HCMC	Ho Chi Minh City
PFIQ-7	Pelvic Floor Impact Questionnaire-7
PFDI-20	Pelvic Floor Distress Inventory-20
POP	Pelvic Organ Prolapse
POPDI-6	Pelvic Organ Prolapse Distress Inventory-6
POP-Q	Pelvic Organ Prolapse Quantification System
POPIQ-7	Pelvic Organ Prolapse Impact Questionnaire-7
QoL	Quality of Life
SD	Standard Deviation
UIQ-7	Urinary Impact Questionnaire-7
UDI-6	Urinary Distress Inventory-6
WHO	World Health Organization
